# ChatGPT and Refugee’s Health: Innovative Solutions for Changing the Game

**DOI:** 10.3389/ijph.2024.1607306

**Published:** 2024-06-11

**Authors:** Shima Jahani, Zahra Dehghanian, Amirhossein Takian

**Affiliations:** ^1^ Multiple Sclerosis Research Center, Neuroscience Institute, Tehran University of Medical Sciences, Tehran, Iran; ^2^ Department of Computer Engineering and Information Technology, Faculty of Engineering, Amirkabir University of Technology, Tehran, Iran; ^3^ Department of Global Health and Public Policy, School of Public Health, Tehran University of Medical Sciences, Tehran, Iran; ^4^ Department of Health Economics and Management, School of Public Health, Tehran University of Medical Sciences, Tehran, Iran

**Keywords:** refugee, refugee health, artificial intelligence (AI), health, ChatGPT

**FIGURE 1:**
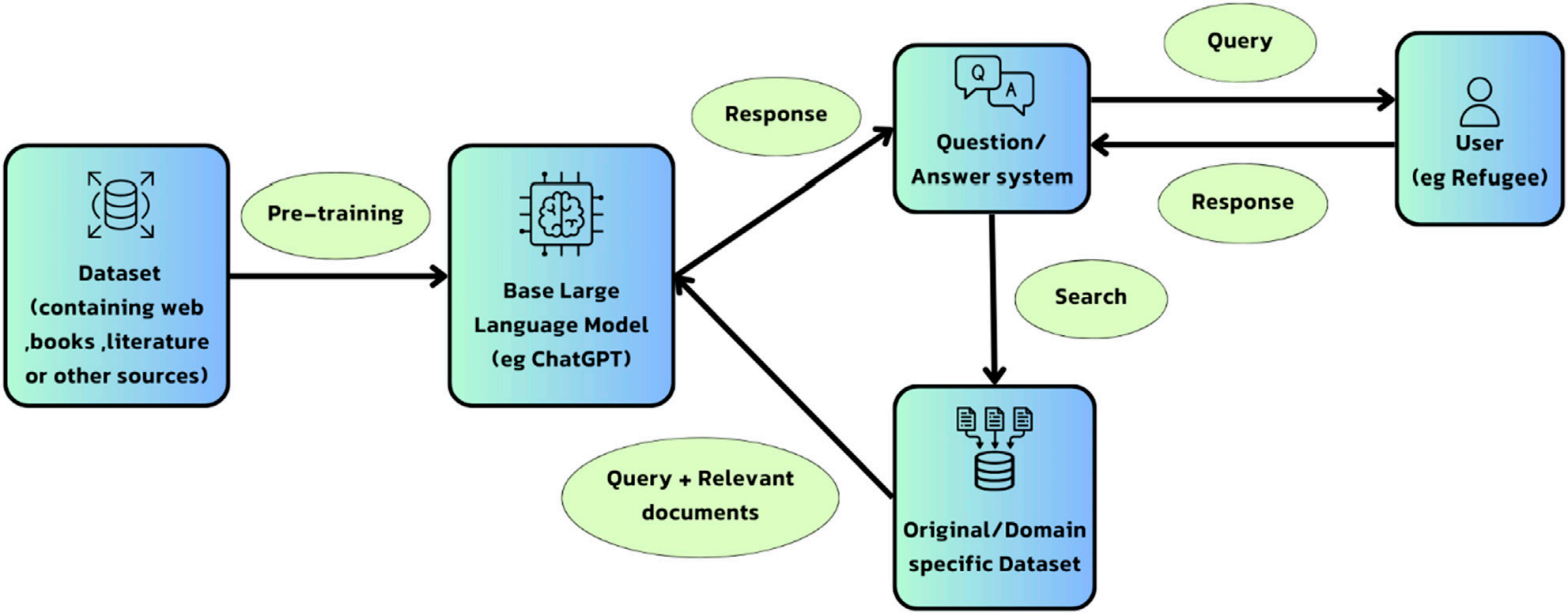
The information flow in a Large Language Model (LLM) by adding Retrieval-Augmented Generation (RAG) (Until 2024, Worldwide).

Refugees, people who were forced to flee their residence due to conflict and persecution, have grown in number significantly in recent years, specifically in low and middle-income countries (LMICs). According to the United Nations’ (UN) High Commissioner for Refugees (UNHCR) at mid 2023, 110 million people have been displaced worldwide, an increase of more than 19 million people compared to the end of 2021; the largest increase in years based on UNHCR’s figures [1].

Refugees face numerous challenges that can have serious impact on their physical and mental health [2]. Despite various international initiatives to assist refugees, i.e., different volunteering resettlement programs and facilities and helps provided by the UN agencies and other organizations, the global refugee crisis has been intensifying in recent years [3]. Hence the crucial need to adapt the application of innovative solutions and programs to respond to refugees’ needs, now more than ever [4]. Among new developments in this field, Germany for instance developed a self-help chatbots for Syrian refugees with posttraumatic stress symptoms [5].

Artificial Intelligence (AI) tools, i.e., ChatGPT present a cutting edge facility in providing efficient and personalized support. ChatGPT is an advanced neuro-linguistic programming (NLP) technology that utilizes the Generative Pre-trained Transformer 3, 3.5 or 4 model. Utilization of ChatGPT and its role in the future of academia, research and healthcare have been hot topics in many publications recently. Table 1 presents some examples of how ChatGPT can change the game for refugee’s support [6]:

**TABLE 1:** Various applications of ChatGPT in refugee’s health (Until 2024, Worldwide).

Support domains	Examples of previously ran programs	How ChatGPT can help refugees
Language support	University of Leicester’s English Language Teaching Unit	1) Providing multilingual translation services;
	Providing free English classes from beginner to more advanced levels [7]	2) Acting as a private teacher for learning foreign languages;
		3) Providing resources in a different roadmap specialized to each one needs and potentials
Community engagement	1) Community sponsorship program: a refugee resettlement program to support refugees in their integration into a new community, being implemented in several countries, i.e., Canada, United Kingdom, and Australia [8]	1) To disseminate information and Provide up-to-date information about local events, resources, and services to aid integration;
	2) Community Centered Integrated Services (CCIS) program: a program to provide support to refugees and migrants such as Trauma-Focused Therapy sessions [9]	2) As a Story Sharing Platforms, it could enable refugees to share their experiences and stories, fostering empathy and connection with local communities
Productivity	UNHCR: provides technical and vocational education and training, which can help refugees learn required skills to enhance their employability [10]	Present learning materials and resources about local customs, laws, societal norms and employment to ease the transition
Mental health support	Mind-Spring, Problem Management Plus, Mental Health First Aid (MHFA), Cognitive-Behavioral Training for Community and Religious Leaders, EmpaTeach, Suicide Prevention Education Program, Teaching Recovery Techniques, Handbook for Teachers of Vietnamese Refugee Students, Psychological First Aid Psychosocial support of volunteers and Community-based protection and Mental health psychological support [11]	1) Providing resources for mind health support;
		2) Medical staff can use this platform to better understand the culture of these populations
		3) Act as a therapist to help them overcome difficult situations they face
Legal assistance	UNHCR legal support:offering legal aid programs to help refugees comprehending their rights and navigate the asylum process [12]	Act as a lawyer or legal assistance for refugees who may be navigating complex legal issues
	Refugee and Immigrant Center for Education and Legal Services (RAICES): offering affordable legal consults to refugees in United States [13]	

Given its significant potential as a life changer act for refugees, meaningful application of ChatGPT requires addressing few limitations:

First, ChatGPT may not understand the diverse background culture of refugees and their specific needs, which could result in ineffective answers and misunderstandings. Therefore, employing strategies for finetuning ChatGPT is necessary to better serve refugees. These strategies include cultural sensitivity training and integration of context-specific knowledge bases. Transfer learning and supervised fine-tuning could facilitate this process.

Second, the availability of ChatGPT to refugees might be limited due to the high cost of internet in low socioeconomic refugee groups. Additionally, the lack of devices and technology literacy may hinder refugees e to employ Chat GPT appropriately. To address this challenge, a low-bandwidth version of ChatGPT application using voice-based interfaces for users with limited literacy or digital skills, and strategies for distributing low-cost or refurbished devices need to be considered [14].

Lastly, since mental health support requires specific understanding of applicants’ condition. Therefore, ChatGPT may not be able to provide the required level of mental health support for refugees. To overcome this condition, the use of Retrieval-Augmented Generation (RAG) technique can ensure the generation of coherent responses that are factually accurate and contextually relevant. The RAG technique (Figure 1) combines the power of language models like ChatGPT with external knowledge retrieval, which enhances the model’s ability to produce contextually relevant information [15, 16].

Another suggestion for enhancing this technology performance is to integrate ChatGPT with refugee support services through Application Programming Interfaces (APIs). This process helps ChatGPT access information from various sources in real-time, for instance using data from social media platforms can enhance the systems understanding of refugees’ perspectives and experiences. Furthermore, to ensure their privacy, blockchain technology can be employed for refugees. Such integration could help them use this chatbot for legal processes.

While utilizing this technology for refugees, it is important to address its potential risks and safety concerns. Data confidentiality and privacy issues are of particular concern, to avoid refugees’ mistrust due to a risk of data leakage. In addition, beyond promises to adhere to open AI’s published privacy policy [17], appropriate safety measures would need to be in place to prevent probable mistakes. Another safety concern for refugees is the lack of empathy, which might lead to misunderstandings, especially in cases with limited literacy. Literature demonstrates risk for generating incorrect, biased or invalid results in ChatGPT [18, 19], which may lead to misguides in refugees. Hence, the crucial need to employ mechanisms to further validate the AI generated results. For instance, pre training datasets with refugees’ specific needs and experiences could increase generated answer’s reliability. Moreover, implementing a framework designed for feedback and improvement, e.g., through reviews from human experts, could enhance the validity of ChatGPT for refugees. Employing processes for Realtime intervention in case of incorrect answer detection, as well as transparency regarding ChatGPT limitations and its potential invalid answers and advising refugees for further human verification, are among mechanisms to build up trust in this technology.

ChatGPT is a potentially valuable AI tool for providing various supports, i.e., healthcare to refugees. However, to achieve impactful changes, it is essential to acknowledge its limitations and supplement the technology with alternative tools.
